# Managing Secondary Findings from Germline Pharmacogenomic Testing

**DOI:** 10.3390/jpm16070390

**Published:** 2026-07-21

**Authors:** Yee Ming Lee, Elizabeth Kearney, David F. Kisor, Christopher L. Farrell

**Affiliations:** 1 Independent Researcher, San Diego, CA 92127, USA; 2 Precision Health Department, UW Health, Madison, WI 53562, USA; ekearney@uwhealth.org; 3 Independent Researcher, Avon Lake, OH 44012, USA; 4 Healthcare Genetics and Genomics Program, School of Nursing, Clemson University, Clemson, SC 29634, USA

**Keywords:** pharmacogenomics, pharmacogenetics, incidental findings, secondary findings, ClinPGx, ClinGen, ACMG, CPIC, genetic counseling

## Abstract

**Background/Objectives**: Germline pharmacogenomics (PGx) testing performed by clinical laboratories and companies can reveal secondary findings (SF) related to gene-disease risk that clinicians must appropriately manage. This study evaluated PGx panel content for potential SF using the Clinical Pharmacogenomics (ClinPGx) resource, the Clinical Genome Resource (ClinGen), and the American College of Medical Genetics and Genomics (ACMG). **Methods**: A cross-sectional review of PGx panels offered by laboratories and companies was assessed for ClinPGx PGx annotation, ClinGen’s gene-disease validity and clinical actionability classifications, Clinical Pharmacogenetics Implementation Consortium (CPIC) incidental finding (IF) comments, and ACMG SF v3.3 inclusion. **Results/Discussion**: Forty-four testing sites provided panel content, yielding 125 genes, alleles, and variants. Of these, 26.4% (33/125) had PGx annotations while 73.6% (92/125) did not. A small subset of genes—*CACNA1S*, *CFTR*, *G6PD*, *LDLR*, *MT-RNR1*, and *RYR1*—had actionable recommendations based on CPIC and ACMG. Additional genes such as *ATM*, *F5*, *ITGB3*, and *SCN1A* may require consultation with genetics professionals. These findings underscore the need for a centralized resource for identifying gene-specific SF from germline PGx testing and guidance on their clinical management. **Conclusions**: PGx panels often include genes with and without established PGx annotations, some of which have potential SF implications. ClinGen’s PGx Working Group, which aims to integrate PGx into the broader context of genomic medicine, may be well-positioned to facilitate the development of a standardized framework for managing potential SF from panel-based PGx testing as the field evolves.

## 1. Introduction

Interest in incorporating germline pharmacogenomics (PGx) in clinical practice continues to grow as PGx can potentially improve drug efficacy and reduce the risk of adverse drug events [1,2,3,4,5]. There are many options to perform PGx testing, including single-gene assays, multigene panels, or sequencing-based approaches. Many clinical laboratories and PGx companies offer targeted gene and variant testing, but genome sequencing is increasingly used due to advances in technology and declining cost.

PGx testing may, however, reveal incidental (IF) or secondary findings (SF) related to disease risk, requiring clinicians to evaluate disease-related genetic information in addition to medication-related PGx results [6]. While PGx examines genetic variants influencing drug response, disease genetics focuses on the pathogenicity of variants and their associated health risks. The American College of Medical Genetics and Genomics (ACMG) and the National Society of Genetic Counselors define IF as unexpected variants identified during genomic analysis, whereas SF refers to variants intentionally analyzed as part of the testing process. In 2013, ACMG published a guideline on reporting IF in clinical exome and genome sequencing [7], but later adopted SF as the standard nomenclature. The ACMG SF list identifies highly penetrant genetic disorders for which interventions can significantly reduce morbidity and mortality [7,8]. The recent ACMG SF v3.3 list includes *CACNA1S* and *RYR1* [9], which are pharmacogenes with Clinical Pharmacogenetics Implementation Consortium (CPIC) guidelines available [10].

The Clinical Pharmacogenomics (ClinPGx) is a centralized PGx resource that integrates information from the Pharmacogenomics Knowledgebase (PharmGKB), CPIC, and the Pharmacogenomics Clinical Annotation Tool (PharmCAT) [11]. It includes clinical PGx guidelines from CPIC and the Dutch Pharmacogenetics Working Group (DPWG) [11,12,13,14,15], and PGx-annotated drug labels from regulatory agencies such as the U.S. Food and Drug Administration (FDA) [16]. Each CPIC guideline includes an IF section that discusses relevant gene-disease and/or variant-disease associations, where applicable.

While ClinPGx focuses on drug-gene and drug-variant associations, the Clinical Genome Resource (ClinGen) is a NIH-funded consortium that evaluates the clinical relevance of genes and variants involved in genetic diseases for use in precision medicine and research [17]. ClinGen contains expert-curated resources such as Gene-Disease Validity and Clinical Actionability databases. The Gene-Disease Validity database classifies evidence linking a gene or variant to monogenic diseases as definitive, strong, moderate, limited, disputed, refuted, or no known association. ClinGen’s Clinical Actionability working group assesses whether genetic findings warrant clinical action by considering outcome severity and likelihood (e.g., penetrance), the effectiveness of available interventions, and the risks, burdens, and acceptability associated with those interventions. Based on these criteria, the group assigns actionability levels of definitive, strong, moderate, or limited.

Certain genes and variants are relevant to both disease risk and drug response. Li et al. evaluated this overlap by reviewing germline genetic annotations from databases such as PharmGKB, CPIC, ClinGen, and Clinically Relevant Variation (ClinVar), and identified 26 genes with strong pathogenic evidence and PGx associations [6]. These include genes such as *CACNA1S* and *RYR1*, which are associated with malignant hyperthermia susceptibility, and *Factor 5* (*F5*), which is associated with thrombosis. The study also identified *SCN1A* and *MTHFR*, which are genes that lack PGx guidelines or FDA PGx label annotations but were linked to drug-response phenotypes. These 26 genes may not be commonly included in testing panels offered by clinical laboratories or PGx companies.

Despite this overlap of genes and variants with disease risk and drug response, there is no formal guidance on how to manage IF and SF from PGx testing. Brown et al. developed a framework for evaluating IF identified in clinical genome sequencing by assessing gene-disease relationships for their clinical significance and actionability using professional guidelines, published literature, and ClinGen resources [18]. This framework was applied to a sequenced cohort of 720 individuals and found 5.1% had IF related to conditions such as cancer predisposition and *G6PD*-related hematological disorders. To our knowledge, no study has examined the extent to which PGx genes, alleles, or variants commonly included in clinical laboratory and commercial PGx panels that offer targeted testing have actionable secondary gene-disease findings.

This study seeks to characterize the prevalence and nature of potential SF identified among PGx genes, alleles, and variants included in panel-based germline PGx testing offered by reference and commercial laboratories and companies. Specifically, we evaluated genes, alleles, and variants for PGx annotations and assessed their gene-disease validity and potential SF actionability using ClinPGx, ClinGen Gene-Disease Validity and Clinical Actionability frameworks, ACMG SF v3.3 criteria, and CPIC IF recommendations.

## 2. Materials and Methods

### 2.1. Identifying Laboratories and Companies Offering PGx Test Panels

We conducted a cross-sectional review of standard reference laboratories, commercial laboratories, and companies offering clinical germline PGx testing between December 2025 and January 2026. Sources included the U.S. NIH Genetic Testing Registry [19] queried using “pharmacogenomic” and “pharmacogenetic” and filtered for U.S.-based laboratories, as well as published literature [20]. Additional sites were identified through querying Google [21] for U.S.-based biotechnology companies that offered PGx testing [22]. PGx gene lists were extracted from company websites and laboratory catalogs using search terms such as “pharmacogenetic,” “pharmacogenomic,” or “genetic testing.” Companies and laboratories that did not list their PGx panel content were contacted for their list. 23&Me was included due to FDA authorization of its direct-to-consumer PGx test. Tests offered for research use or those focused on somatic mutations were excluded. For sites offering multiple panels, duplicate gene lists were consolidated into a single list per site.

### 2.2. Evaluating Genes for PGx Annotation

Most laboratories and companies reported panel content at the gene level, while some reported specific variants. Data were harmonized at the gene level for frequency analyses, except for *HLA* alleles and the *CYP2C* cluster variant (rs 12777823), which were analyzed at the allele and variant levels, respectively. The identified genes, alleles, and variants were evaluated for PGx annotations by CPIC, DPWG, and FDA drug labels using ClinPGx. For drug-gene pairs with CPIC guidelines, CPIC IF comments were reviewed to identify actionable disease-risk information. Drug-gene pairs evaluated by CPIC or DPWG and designated as having “no recommendation” were classified as having no PGx annotation. For FDA-label PGx annotations, drug-gene pairs assigned to one of the following ClinPGx’s PGx levels were included for analysis: testing required, testing recommended, actionable PGx, or informative PGx.

### 2.3. Evaluating Genes for Gene-Disease Relationships and SF Actionability

PGx genes, alleles, and variants were evaluated for gene-disease relationships and SF actionability using ClinGen Gene-Disease Validity and Clinical Actionability frameworks, ACMG SF v3.3 inclusion, and CPIC IF-recommended actions [9,23]. Only genes with moderate, strong, or definitive ClinGen Gene-Disease Validity and Clinical Actionability ratings were included in the analysis.

## 3. Results

### 3.1. PGx Laboratories and Companies

**Figure 1:**
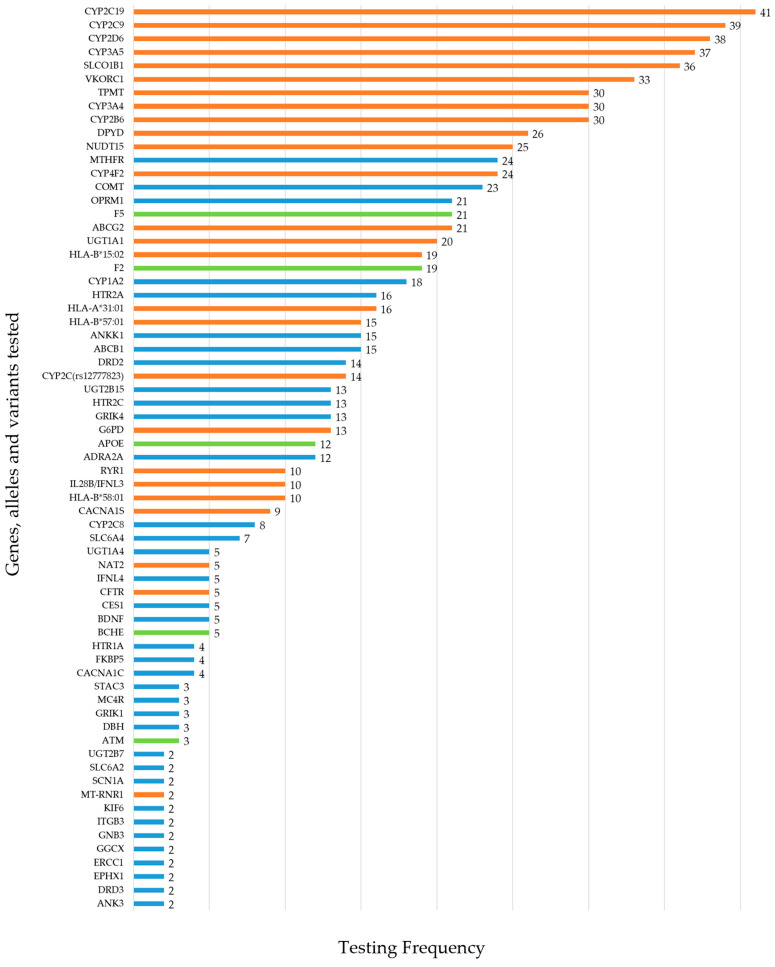
Testing frequency of genes, alleles and genetic variants reported by at least 2 out of 44 sites. Bar colors indicate pharmacogenomics (PGx) annotation source: orange represents Clinical Pharmacogenetics Implementation Consortium (CPIC), Dutch Pharmacogenetics Working Group (DPWG) and Food and Drug Administration (FDA) annotation; green represents FDA-only annotation; and blue represents no PGx annotation.

A total of 104 laboratories and companies (collectively referred to as “sites”) were identified through our search (Supplement Table S1). Of these, 33 did not offer PGx panel testing, 17 were no longer in operation (e.g., closed, acquired, or rebranded), and PGx content from 10 sites was unavailable at the time of final analysis. Forty-four sites had their PGx panel content available, yielding 125 genes, alleles, and variants for analysis. Figure 1 shows the testing frequency of these genes, alleles, and variants offered by at least two sites in descending order. Their corresponding PGx annotations (CPIC, DPWG, FDA label) and gene-disease annotations (ClinGen Gene-Disease Validity, Clinical Actionability, and ACMG SF) are shown in Supplement Table S2.

### 3.2. Testing Frequency and PGx Annotation

The top ten genes, alleles, and variants tested in descending order of frequency were *CYP2C19*, *CYP2C9*, *CYP2D6*, *CYP3A5*, *SLCO1B1*, *VKORC1*, followed by a three-way tie among *TPMT*, *CYP3A4*, and *CYP2B6*, *DPYD*, *NUDT15*, and a two-way tie among *MTHFR* and *CYP4F2*. Among these, 92.3% (12/13) had PGx annotations from CPIC, DPWG, or FDA label, with the exception of *MTHFR*, for which DPWG reported no recommendation for *MTHFR*-methotrexate drug-gene pair [24].

**Figure 2:**
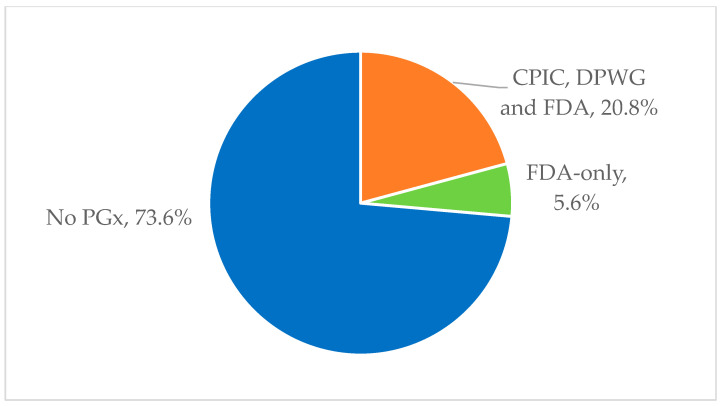
Pharmacogenomic annotation of the 125 genes, alleles, and variants reported by the testing laboratories and companies.

Of the 125 genes, alleles, and variants analyzed, 26.4% (33/125) had PGx annotations—20.8% from CPIC, DPWG, and FDA label, and 5.6% from FDA-only (Table 1, Figure 2). The remaining 73.6% (92/125) had no PGx annotation.

### 3.3. Genes with PGx Annotation and Their Gene-Disease Relationships and SF Actionability

Table 2 shows the 33 genes, alleles, and variants with PGx annotations. Of these, 12 (36.4%) had CPIC IF comments: *ABCG2*, *CFTR*, *DPYD*, *G6PD*, HLA-B*57:01, *IFNL3*, *MT-RNR1*, *NAT2*, *RYR1*, *SLCO1B1*, *UGT1A1*, and *VKORC1* (Table 1). Review of the CPIC IF comments showed that most were informational; however, four had specific recommendations, including referral to the genetics service for *CFTR*, *G6PD*, and *MT-RNR1*, and referral to a neuromuscular specialist for *RYR1* (Table 2). Testing frequency for these four genes varied across sites, ranging from 30% for *G6PD* to 5% for *MT-RNR1*.

Eight genes (24.2%) had moderate-to-definitive ClinGen Gene-Disease Validity annotations: *ATM*, *CACNA1S*, *F2*, *F5*, *G6PD*, *MT-RNR1*, *RYR1*, *and VKORC1* (Table 2). Among these, five genes (15.2%) had moderate-to-definitive ClinGen clinical actionability ratings: *ATM*, *CACNA1S*, *F5*, *G6PD*, and *RYR1*. Notably, *CACNA1S* and *RYR1* are included on the ACMG SF list and are associated with malignant hyperthermia susceptibility [10], warranting referral to genetic services. Both genes were tested by at least 20% of the sites. *F5* was tested in nearly half of the sites (48%), with its ClinGen Clinical Actionability rating varying by F5 deficiency status and F5 Leiden genotype. Although *ATM* had moderate clinical actionability in adults and was associated with an increased risk of breast cancer, it was tested at fewer than 10% of the sites.

### 3.4. Genes with No PGx Annotation and Their Gene-Disease Relationships and SF Actionability

Among the 92 genes with no PGx annotations, 15 (16.3%) had moderate-to-definitive Gene-Disease Validity classifications (Table 1). Of these genes, only three—*ITGB3*, *SCN1A*, and *LDLR*—had moderate-to-definitive clinical actionability ratings (Table 3). *LDLR* is included in the ACMG SF v3.3 list due to its association with familial hypercholesterolemia. All three genes were tested in no more than 5% of the sites.

## 4. Discussion

This study evaluated the current landscape of germline PGx panel testing and characterized genes and variants with and without PGx annotation for potential SF. PGx panel content was available from 44 sites, yielding 125 genes, alleles, and variants for analysis. The study identified a limited set of genes with potentially actionable SF, with actionability and corresponding management varying by specific variant, phenotype, and clinical condition. These include *CACNA1S*, *CFTR*, *G6PD*, *LDLR*, *MT-RNR1*, and *RYR1*, while other genes—*ATM*, *F5*, *ITGB3*, and *SCN1A*—may warrant further consultation with genetic services. Together, these findings highlight the need for a structured framework to guide clinicians who order panel-based PGx tests or interpret patient-provided PGx reports in identifying and managing potential SF. This need is likely to intensify as new PGx testing laboratories and companies emerge, panel content evolves, and increasing heterogeneity across PGx panels expands the potential for identifying additional SF.

Among PGx-annotated genes, alleles, and variants, CPIC IF comments served as a reliable resource on the potential action needed. This study found actionable recommendations for *CFTR*, *G6PD*, *MT-RNR1*, and *RYR1* that involved referral to genetic services or appropriate specialists, depending on the specific gene involved. There were two genes—*ATM* and *F5*—with FDA-only PGx annotations and no CPIC guidelines. *F5* had differing clinical actionability based on carrier status, with limited actionability in heterozygous adults and moderate actionability in homozygous adults. Although *F5* was tested in nearly half of the sites evaluated (48%), the prevalence of *F5* deficiency or *F5* Leiden carriers may vary across biogeographic groups [25]. Additionally, genomic databases and the design of targeted commercial PGx panels remain largely Eurocentric, which can affect both PGx detection rates and the interpretation of SF [26]. For example, *G6PD* exhibits substantial variation in allele distribution across populations of African Mediterranean, Asian, or European ancestry [27]. Nevertheless, clinicians need to know how to manage potential SF when identified [28].

There were three genes with no PGx annotations—*ITGB3*, *SCN1A*, and *LDLR.* Although these were infrequently tested, clinicians should be aware that *ITGB3* has strong ClinGen clinical actionability in both adult and pediatric populations, indicating that a consult with genetic services may be needed. For other genes with moderate ClinGen clinical actionability, such as *ATM* (FDA label PGx annotation) and *SCN1A* (no PGx annotation), consultation with genetic services may help guide whether a higher actionability threshold (e.g., strong or definitive) should guide decisions about returning these results as potential SF.

Clinicians should also be familiar with the ACMG SF v3.3 list when selecting PGx panels or reviewing PGx reports, as variants in *CACNA1S*, *RYR1*, and *LDLR* require appropriate follow-up when identified. *MTHFR* was frequently tested (55%) across sites despite lacking PGx annotation. Although Li et al. reported *MTHFR* as having strong PGx and pathogenic support for *MTHFR*-methotrexate association, DPWG guidelines published later assigned “no PGx recommendation” following their review of the evidence [22]. ClinGen assigned no clinical actionability rating to *MTHFR*, and ACMG has advised against MTHFR testing [29]. This is an example of a commercial panel testing disconnect where commercial PGx panels may not be updated to reflect the latest scientific findings. Given the continued interest in *MTHFR*, clinicians will need to monitor for updates to PGx guidelines and ClinGen, as new evidence may prompt reevaluation of its actionability.

This study also highlights a key technical barrier associated with PGx panel implementation: the lack of standardization of PGx panels [30]. Variants tested for a given gene may differ across laboratories and commercial PGx companies. To address this, the US-based Association of Molecular Pathology has issued consensus guidelines that specify a minimum set of variants (Tier 1) for inclusion in clinical PGx genotyping assays [31,32,33,34,35,36]. Additionally, because PGx testing focuses on variants associated with drug response, PGx panels may not capture or report all disease-relevant pathogenetic variants relevant to ACMG SF or ClinGen disease actionability frameworks.

The study findings also underscore the need for a consensus list of potential SF associated with germline PGx testing, along with a centralized resource to guide clinical management. While this study evaluated the potential SF clinical action using ClinGen and ClinPGx, notable differences exist between these resources. These include differences in terminology used to describe validity and actionability. For example, ClinGen evaluates clinical validity and actionability separately, while ClinPGx considers these domains collectively to support PGx clinical utility [37]. To bridge this gap, ClinGen reestablished the ClinGen Pharmacogenomics Working Group (ClinGen PGxWG) in 2022 to integrate PGx into ClinGen’s existing frameworks. The PGxWG is developing standardized methods to assess gene-drug pairs for clinical validity and actionability, and may play a key role in formulating a systematic approach for assessing genes for potential SF.

Currently, SF is not consistently reported in PGx reports [38], and may be embedded within technical appendices of laboratory reports. This lack of standardized reporting adds complexity for clinicians who may not be familiar with identifying SF from germline PGx testing. Some clinicians also may not feel comfortable or confident in interpreting SF compared to genetics-trained professionals, such as genetic counselors [39]. Although some institutions have genetic counselors in their Personalized Medicine clinics [40,41,42], this model is not universally available. To circumvent this, clinicians without local genetics support may use the National Society of Genetic Counselors’ “Find a Genetic Counselor” directory (www.nsgc.org) and apply search filters such as telehealth, location of interest, and personalized genomic medicine to find genetic counselor services.

Of note, this study did not discuss the ethical and practical challenges of managing potential SF, including developing a plan for returning actionable SF, and considering patient preferences for receiving potentially actionable SF [38,43].

There are several limitations to this study. First, clinical laboratories and PGx companies may have changed their PGx testing panels since this study was conducted, and new testing sites may have emerged. Second, the analyses focused on PGx panels and excluded single-gene PGx tests. The study did not assess variant classification practices and evaluate whether PGx panels captured pathogenic or likely pathogenic variants. Third, as this study evaluated PGx testing sites within the U.S., its findings may not be generalizable to other countries. Fourth, the study did not evaluate confirmatory testing recommendations. Fifth, the study did not review all guidelines reflecting the clinical actionability of genomic variants, including those from the National Comprehensive Cancer Network and the Centers for Disease Control and Prevention’s Tier 1 Population Screening Recommendations. Finally, the study did not conduct a comprehensive review of PGx panels offered by academic medical centers.

## 5. Conclusions

PGx panels frequently include genes with and without established PGx annotations, some of which have SF implications. Although this study identified a limited number of genes with actionable SF, the findings highlight a broader need for a centralized, standardized framework to support the identification and management of SF arising from panel-based germline PGx testing. This need will grow as PGx testing expands and panel content evolves and becomes more heterogeneous. ClinGen’s PGxWG may be well-positioned to facilitate the development of consensus-based guidance for SF management as the field of genomic medicine advances.

## Figures and Tables

**Table 1:** Distribution of 125 genes, alleles, and variants evaluated based on pharmacogenomic-annotation status, annotation source, CPIC Incidental Finding comment, ClinGen Gene-Disease Validity and Clinical Actionability classification, and ACMG Secondary Finding v3.3 inclusion.

PGx Annotation Status and Source		CPIC IF Comment	ClinGen Gene-Disease Validity ^a^	ClinGen Clinical Actionability ^b^	ACMG SF v3.3
PGx annotation N = 33	CPIC, DPWG, and FDA N = 26 *ABCG2*, *CACNA1S*, *CFTR*, *CYP2B6*, *CYP2C19*, *CYP2C9*, *CYP2D6*, *CYP3A4*, *CYP3A5*, *CYP4F2*, *CYP2C (rs12777823)*, *DPYD*, *G6PD*, HLA-A*31:01, HLA-B*15:02, HLA-B*57:01, HLA-B*58:01, *IFNL3*, *MT-RNR1*, *NAT2*, *NUDT15*, *RYR1*, *SLCO1B1*, *TPMT*, *UGT1A1*, *VKORC1*	N = 12 *ABCG2*, *CFTR*, *DPYD*, *G6PD*, HLA-B*57:01, *IFNL3*, *MT-RNR1*, *NAT2*, *RYR1*, *SLCO1B1*, *UGT1A1*, *VKORC1*	N = 5 *CACNA1S*, *G6PD*, *MT-RNR1*, *RYR1*, *VKORC1*	N = 3 *CACNA1S. G6PD*, *RYR1*	N = 2 *CACNA1S*, *RYR1*
	FDA-only N = 7 *APOE*, *ATM*, *BCHE*, *F2*, *F5*, *HLA-DQA1*, *HLA-DRB1*	-	N = 3 *ATM*, *F2*, *F5*	N = 2 *F5*, *ATM*	N = 0
No PGx annotation N = 92	-	-	N = 15 *ACE*, *ANK3*, *CACNA1C*, *F13A1*, *GGCX*, *GRIN2B*, *ITGB3*, *ITPA*, *LDLR*, *MTHFR*, *POR*, *SCN1A*, *SLC1A2*, *STAC3*, *TH*	N = 3 *ITGB3*, *LDLR*, *SCN1A*	N = 1 *LDLR*

ACMG: American College of Medical Genetics and Genomics; CPIC: Clinical Pharmacogenetics Implementation Consortium; DPWG: Dutch Pharmacogenetics Working Group; FDA: Food and Drug Administration; IF: incidental finding; PGx: pharmacogenomics; SF: secondary finding. ^a^ ClinGen gene-disease validity classification that is moderate, strong, or definitive; ^b^ ClinGen clinical actionability classification that is moderate, strong, or definitive.

**Table 2:** Pharmacogenomic (PGx)-annotated genes, alleles, and variants (N = 33) in descending order of testing frequency stratified by CPIC Incidental Finding comment, ACMG SF inclusion, ClinGen Gene-disease Validity level, ClinGen Clinical Actionability level, and summary of clinical action.

Genes, Alleles and Variants	Testing Frequency out of 44 Sites (N,%)	CPIC IF Comment Available	ACMG SF Inclusion	ClinGen Gene-Disease Validity ^a^ Level	ClinGen Clinical Actionability ^b^ Level	Summary of Clinical Action
*CYP2C19*	41 (93)					
*CYP2C9*	39 (89)					
*CYP2D6*	38 (86)					
*CYP3A5*	37 (84)					
*SLCO1B1*	36 (82)	X				
*VKORC1*	33 (75)	X		Moderate		
*TPMT*	30 (68)					
*CYP3A4*	30 (68)					
*CYP2B6*	30 (68)					
*DPYD*	26 (59)	X				
*NUDT15*	25 (57)					
*CYP4F2*	24 (55)					
*ABCG2*	21 (48)	X				
*F5*	21 (48)			Definitive	*F5* deficiency (Strong-adult), *F5* Leiden homozygous (Moderate-adult)	
*UGT1A1*	20 (45)	X				
*F2*	19 (43)			Definitive		
HLA-B*15:02	19 (43)					
HLA-A*31:01	16 (36)					
HLA-B*57:01	15 (34)	X				
*CYP2C* *rs 12777823*	14 (32)					
*G6PD*	13 (30)	X		Definitive	Moderate-adult and pediatric	*G6PD* deficiency is associated with an increased risk for hemolytic anemia under certain conditions. Refer to genetic services
*APOE*	12 (27)					
HLA-B*58:01	10 (23)					
*IFNL3*	10 (23)	X				
*RYR1*	10 (23)	X	X	Definitive	Strong-adult and pediatric	*RYR1* variant is associated with malignant hyperthermia susceptibility. Refer to neuromuscular specialist and genetic services
*CACNA1S*	9 (20)		X	Moderate	Strong-adult and pediatric	*CACNA1S* variant is associated with malignant hyperthermia susceptibility. Refer to genetic services
*BCHE*	5 (11)					
*CFTR*	5 (11)	X				Refer to genetic services
*NAT2*	5 (11)	X				
*ATM*	3 (7)			Definitive	Moderate-adult	
*MT-RNR1*	2 (5)	X		Definitive		*MT-RNR1* variant is associated with aminoglycoside-induced hearing loss. Refer to genetic services
*HLA-DQA1*	1 (2)					
*HLA-DRB1*	1 (2)					

ACMG: American College of Medical Genetics and Genomics, CPIC: Clinical Pharmacogenetics Implementation Consortium, IF: incidental finding, SF: secondary finding; ^a^ ClinGen Gene-disease Validity level that is moderate, strong, or definitive; ^b^ ClinGen Clinical Actionability level that is moderate, strong, or definitive; X indicates inclusion in the specified category.

**Table 3:** Selected genes, alleles, and variants with no pharmacogenomic (PGx) annotation (N = 3) listed in descending order of testing frequency, stratified by CPIC Incidental Finding comment, ACMG SF inclusion, ClinGen Gene-disease Validity level, ClinGen Clinical Actionability level, and summary of clinical action.

Genes, Alleles and Variants	Testing Frequency out of 44 Sites (N,%)	CPIC IF Comment	ACMG SF Inclusion	ClinGen Gene-Disease Validity ^a^ Level	ClinGen Clinical Actionability ^b^ Level	Summary of Clinical Action
*ITGB3*	2 (5)			Definitive	Strong-adult and pediatric	
*SCN1A*	2 (5)			Definitive (epilepsy). Moderate (familial hemiplegic migraine)	Moderate–pediatric	
*LDLR*	1 (2)		X		Heterozygous familial hypercholesterolemia (Definitive-adult; strong-pediatric). Homozygous familial hypercholesterolemia (Strong–adult and pediatric)	*LDLR* variant associated with familial hypercholesterolemia. Refer to genetics service

ACMG: American College of Medical Genetics and Genomics, CPIC: Clinical Pharmacogenetics Implementation Consortium, IF: incidental finding, SF: secondary finding; ^a^ ClinGen Gene-disease Validity level that is moderate, strong, or definitive; ^b^ ClinGen Clinical Actionability level that is moderate, strong, or definitive; X indicates inclusion in the specified category.

## Data Availability

The original contributions presented in this study are included in the article/Supplementary Materials. Further inquiries can be directed to the corresponding authors.

## Supplementary Materials

The following supporting information can be downloaded at: https://www.mdpi.com/article/10.3390/jpm16070390/s1, Table S1: Clinical laboratories and companies evaluated for PGx panel content; Table S2: Genes and their testing frequency, PGx annotations (CPIC, DPWG, FDA, ACMG), and ClinGen classification (Gene-Disease Validity, Clinical Actionability).
