# Supplementary material for: Managing Secondary Findings from Germline Pharmacogenomic Testing
Source: J Pers Med. 2026 Jul 21;16(7):390. doi: 10.3390/jpm16070390 (PMC13412467; doi:10.3390/jpm16070390)
Supplement: Supplementary file 1 [file jpm-16-00390-s001.zip › jpm-4329999-supplementary.pdf]

Supplement Table S1: Clinical laboratories and companies evaluated for PGx panel content

No	Company	Website
1	AccessDx	https://www.accessdxlab.com/pharmacogenomics/
2	23&Me	https://www.23andme.com/test-info/pharmacogenetics/?srsltid=AfmBOopp2JkloW-HvrvuGuZfnOD0VIvYQaSk2Hy4jIXhaq-zd3Zw2Sps
3	3x4 Genetics	https://3x4genetics.com/
4	Admera Health PGxOne Plus	https://www.ncbi.nlm.nih.gov/gtr/tests/567653/#clinical-information
5	Advantage diagnostics clinical laboratory	https://advantagediagnosticlaboratory.com/services/genetics/pgx/
6	Aegis Sciences Corporation	https://www.aegislabs.com/
7	Agena Bioscience	https://www.agenabio.com/applications/panel/veridose-core-panel/
8	Alcala Labs	https://www.alcalalabs.com/
9	All of Us Research Program	https://support.researchallofus.org/hc/en-us/articles/36415867566996-All-of-Us-Pharmacogenomics-Star-Allele-Calling
10	Ally clinical diagnostics	allyclinicaldiagnostics.com
11	Ariel Precision Medicine	https://arielmedicine.com/
12	ARUP lab	https://www.aruplab.com/
13	Assurance medical testing	https://www.assurancemedtesting.com/drug-testing
14	Bio Genetisys	https://biogenetisysinc.com/
15	Biocerna	www.biocerna.com
16	BioGenetics	https://bio-genetics.com/pharmacogenetics/
17	Biron	https://dna.biron.com/mental-health-adhd
18	Boston Heart Diagnostics	https://bostonheartdiagnostics.com/boston-heart-test-menu/
19	Castle Biosciences	https://castlebiosciences.com/tests
20	Childrens hospital of Philadelphia	chrome-extension://efaidnbmnnnibpcajpcglclefindmkaj/https://media.chop.edu/data/files/pdfs/molecular-genetics-lab-test-requisition-form.pdf
21	Cincinnati Children's hospital	https://www.cincinnatichildrens.org/service/g/genetic-pharmacology/drugs-tested
22	ClarityxDNA	https://clarityxdna.com/
23	Color	https://support.color.com/en/articles/2393666-what-is-the-medication-response-genetic-test
24	Compliance Advantage	http://callaboratory.com
25	CoreBiolabs	https://corebiolabs.com/
26	Corielle institute	https://www.coriell.org/
27	Cquentia	www.cquentia.com
28	Cygenex	https://cygenex.com/medicine-management/
29	Dynamic DNA Laboratories	https://dynamicdnalabs.com/collections/personalized-medicine

30	Epinex diagnostics laboratories	www.epinex.com
31	Eurofins Genomics	https://eurofinsgenomics.com/en/home/
32	Exceltox laboratories	https://www.exceltox.com/pharmacogenomic-testing/
33	Express Gene	https://expressgene.us/product/express-gene-comprehensive-pharmacogenomics-pgx-provider/
34	Firefly diagnostics	www.thegaptest.com
35	Firmalab	https://firmalab.com/pharmacogenetics/
36	Fulgent	https://www.fulgentgenetics.com/pgx-comprehensive
37	Geisinger Medical Laboratories	https://www.geisingermedicallabs.com/catalog/
38	GeneAlgin	www.genealign.com
39	GeneDx	https://www.genedx.com/
40	Genelex	https://www.genelex.com/test-menu/
41	Genemarkers	https://genemarkersllc.com/precision-medicine-solutions/
42	Genepath	https://www.genepathdx.com/
43	Genesight	https://genesight.com/
44	Genetic Technological Innovations	https://www.gtilaboratories.com/services
45	Genetworx	https://genetworx.com/effective-rx/employers/
46	Genomind	https://genomind.com/solutions/pharmacogenetic-testing/
47	Genoscientific	http://genoscientific.com/
48	Ghome Diagnostics	https://gnomesciences.com/
49	Gravity Diagnostics	https://gravitydiagnostics.com/pharmacogenetics-testing/
50	Gulfstream diagnostics	http://www.gulfdiagnostics.com
51	Health Care Providers Laboratory	www.healthcareproviderslaboratory.us
52	Helix	https://www.helix.com/test-catalog/pharmacogenomics
53	Highline labs	https:highline1.com
54	Indiana University PGx Pharmacogenomics Genotyping Panel	https://medicine.iu.edu/genetics/genetic-testing-laboratories/test-directory/pgx-genotyping-panel
55	Integralabs	http://www.integralaboratories.com/
56	Invitae	https://www.invitae.com/providers/test-catalog/test-03285
57	Iowa Institute of Human Genetics	https://humangenetics.medicine.uiowa.edu/
58	Iversion Genetics	http://iversiongenetics.com/
59	Kailos	https://www.kailosgenetics.com/kailos-lab/inspexiontm-pharmacogenomic-testing
60	Lab Express	https://www.labexpresscorp.com/
61	Lab Genomics	https://labgenomic.com/drug-metabolism/
62	Lab Source	http:labsource.net

63	LabCorp	chrome-extension://efaidnbmnnnibpcajpcglclefindmkaj/https://www.labcorp.com/assets-media/2958
64	Labtest Diagnostics	http://www.labtestdiagnostics.com/
65	Luxor Scientific	https://luxorscientific.org/test-menu/
66	Mass General Brigham	https://www.massgeneralbrigham.org/en/research-and-innovation/centers-and-programs/personalized-medicine/molecular-medicine/tests/genome#accordion-c83317121b-item-8def4036a1
67	Mayo Clinic Laboratories	https://www.mayocliniclabs.com/
68	MD diagnostics	https://www.md-diagnostics.com/molecular-testing
69	Medicover Genetics	https://medicover-genetics.com/our-genetic-tests/pharmacogenomics/
70	Millenium Health laboratories	https://www.millenniumhealth.com/
71	Molecular testing labs	https://moleculartestinglabs.com/testing-menu/
72	Navis Clinical Laboratories	https://navisclinical.com/pharmacogenetic-testing/
73	Neurokaire	https://www.neurokaire.com/
74	Next Molecular Analytics	https://www.nextmolecular.com/pharmacogenomics
75	OneOme	https://oneome.com/rightmed-test/
76	Oregon Health and Science University	https://www.ohsu.edu/lab-services/test-directory
77	Pathway Genomics	www.pathway.com
78	Patients Choice Laboratories	https://www.pclabsdx.com/pgx
79	Personalized Medicine Care Diagnostics	https://pmcdx.com/pharmacogenomics-pgx-panels/
80	PersonalizedDxLabs	www.personalizedxlabs.com
81	Phenomics Health	https://www.phenomicshealth.com/clinical-products-2/
82	Pinnacle Lab Service	https://www.pinnaclelabservices.net/
83	PlexusDx	https://plexusdx.com/
84	Premier Medical	https://premiermed.com/services/laboratory/
85	Primex Clinical Laboratories	https://primexlab.com/tests/
86	Prometheus laboratories	https://prometheuslabs.com/
87	Proove Biosciences	www.proove.com
88	Quantigen Diagnostics	https://qualigendiagnostics.com/
89	Quest diagnostics	https://www.questdiagnostics.com/healthcare-professionals/about-our-tests/genetics/pharmacogenomics
90	RPRD diagnostics	https://www.rprdx.com/testing/whole-pharmacogenomics-scan-wps/
91	Rxight	https://www.rxight.com/
92	Sanford Imagenetics	https://imagenetics.sanfordhealth.org/pharmacogenomics/
93	Sema4	https://sema4genomics.com
94	Solaris	https://solarisdx.com/test/pharmacogenomics-pgx/
95	SOLVD Health	https://solvdhealth.com/prescript/

96	SOLVDH Health (former Prescient Medicine Personalized Dx labs)	https://solvdhealth.com/prescript/
97	Sorenson Genomics	https://sorensongenomics.com/
98	Sunrise Medical Laboratories	https://www.sunriselab.com/
99	Tempus	https://www.tempus.com/neurology-psychiatry/
100	University of California San Francisco Health Clinical Laboratories	https://www.testmenu.com/UCSFClinLab/Tests/811978 (PMID 39665424)
101	University of Florida MyRx	https://ufhealth.org/myrx/for-providers
102	Variantyx	https://www.variantyx.com/products-services/genomic-unity-pharmacogenomics/
103	Xact laboratories	https://xactlaboratories.com/
104	You Script	https://youscript.com/

Supplement Table S2: Genes and their testing frequency, PGx annotations (CPIC, DPWG, FDA, ACMG) and ClinGen classification (Gene-Disease Validity, Clinical Actionability)

Gene	Testing Frequency	% of 44 sites	CPIC or DPWG Guideline	CPIC Incidental Finding Comment	FDA Label PGx Annotation	ACMG SF v3.3 Annotation	ClinGen Gene-Disease Validity Classification	ClinGen Clinical Actionability Classification
CYP2C19	41	93%	X	-	X	-	-	-
CYP2C9	39	89%	X	-	X	-	-	-
CYP2D6	38	86%	X	-	X	-	-	-
CYP3A5	37	84%	X	-	-	-	-	-
SLCO1B1	36	82%	X	Complete SLCO1B1 and SLCO1B3 deficiency is associated with Rotor syndrome.	X	-	-	-
VKORC1	33	75%	X	Homozygosity for rare coding mutations in VKORC1 are a known cause of combined deficiency of vitamin K dependent clotting factors-2 (VKCFD2), which is a rare and potentially fatal bleeding disorder	X	-	VKORC1 is associated with vitamin K-dependent clotting factor deficiency (moderate)	-
CYP2B6	30	68%	X	-	X	-	-	-
CYP3A4	30	68%	X	-	-	-	-	-
TPMT	30	68%	X	-	X	-	-	-

DPYD	26	59%	X	Individuals who harbor one copy of a no function DPYD variant can be considered to have carrier status for an inborn error of metabolism and consideration should be given to its potential effects on offspring. Patients homozygous for inactivating variants of DPYD have complete dihydropyrimidine dehydrogenase deficiency, a clinically heterogeneous autosomal recessive disorder of pyrimidine metabolism that shows wide variability of clinical presentations, ranging from no symptoms to severe convulsive disorders with motor and mental retardation	X	-	-	-
NUDT15	25	57%	X	-	X	-	-	-
CYP4F2	24	55%	X	-	-	-	-	-
MTHFR	24	55%	-	-	-	-	Homocystinuria due to MTHFR deficiency (definitive)	-
COMT	23	52%	-	-	-	-	-	-
ABCG2	21	48%	X	Genome- wide association studies reveal that ABCG2 variants influence serum uric acid levels, risk for gout, and response to the antigout medication, allopurinol. In addition, null ABCG2 expression is associated with the Junior blood group, which determines presence of the Jr(a) antigen.	-	-	-	-

F5	21	48%	-	-	X	-	F5 deficiency is associated with thrombophilia due to activated protein C resistance (definitive)	F5 deficiency (Adult-Strong actionability), F5 Leiden Heterozygous (Adult - Limited actionability, Pediatric - Insufficient evidence), F5 Leiden homozygous (Adult-Moderate actionability)
OPRM1	21	48%	-	-	-	-	-	-
UGT1A1	20	45%	X	Reduced hepatic UGT1A1 activity to 30% of normal is a hallmark of Gilbert syndrome, a benign condition characterized by mild unconjugated hyperbilirubinemia.4 Individuals with Gilbert syndrome may experience transient elevations in unconjugated plasma bilirubin in response to various triggers (e.g., fasting, infection, or medications). Genotypes most commonly implicated in Gilbert syndrome are UGT1A1*28/*28 and UGT1A1*6/*6.	X	-	-	-
F2	19	43%	-	-	X	-	Thrombophilia due to thrombin defect (definitive)	-
*HLA-B*15:02*	19	43%	X	-	X	-	-	-
CYP1A2	18	41%	-	-	-	-	-	-
*HLA-A*31:01*	16	36%	X	-	X	-	-	-
HTR2A	16	36%	-	-	-	-	-	-
ABCB1	15	34%	-	-	-	-	-	-
ANKK1	15	34%	-	-	-	-	-	-

*HLA-B*57:01*	15	34%	X	HLA-B*57:01 has also been shown to be overrepresented in HIV long-term nonprogressors, the small group of HIV positive patients in whom, despite the absence of antiretroviral therapy, the condition does not progress to AIDS. This suggests that HLA-B*57:01 in some way confers a host immune response that is better able to control the virus. In addition, HLA-B*57:01 has been associated with a lower viral load set point in Caucasians; similar associations, with lower viral loads, have been observed in African Americans with the closely related allele HLA-B*57:03.	X	-	-	-
CYP2C(rs12777823)	14	32%	X	-	-	-	-	-
DRD2	14	32%	-	-	-	-	-	-

G6PD	13	30%	X	Patients with G6PD deficiency should be advised that they are at an increased risk of hemolysis after exposure to fava beans or to high-risk drugs or chemicals, and that it is recommended to avoid such substances. Other conditions, such as infection, hyperuricemia, and sepsis that lead to generation of activated oxygen species, also place the patient at risk of A=acute hemolytic anemia. Furthermore, because the G6PD gene is located on the X chromosome, self-identified males who have a G6PD diplotype indicating the presence of two G6PD alleles may have an inherited sex chromosome disorder such as Klinefelter syndrome. This syndrome occurs in ~ 1 in 600 persons assigned male at birth, and there are possible medical interventions that may be indicated once that diagnosis is confirmed. Consideration for involvement of genetic counselors and procedures to confirm the diagnosis of Klinefelter syndrome should be in place for those who routinely test G6PD genotype.	X	-	G6PD deficiency associated with non spherocytic hemolytic anemia (definitive)	G6PD deficiency (Adult and Pediatric - Moderate actionability)
GRIK4	13	30%	-	-	-	-	-	-
HTR2C	13	30%	-	-	-	-	-	-
UGT2B15	13	30%	-	-	-	-	-	-
ADRA2A	12	27%	-	-	-	-	-	-
APOE	12	27%	-	-	X	-	-	-
*HLA-B*58:01*	10	23%	X	-	X	-	-	-

IL28B/IFNL 3	10	23%	X	The IFNL3 rs12979860 polymorphism has also been linked to HCV-induced hepatocellular carcinoma and graft fibrosis, allergic disease in children, liver fibrosis, viral cirrhosis due to HCV, and greater likelihood of HCV persistence, particularly in HCV genotypes 1 and 4. The favorable rs12979860 CC genotype is associated with lower frequency of hepatic steatosis in patients with chronic HCV. Carriers of the T allele of this variant have also been found to have increased susceptibility to chronic hepatitis B virus (HBV) infection and hepatocellular carcinoma as compared with noncarriers.	X	-	-	-

RYR1	10	23%	X	Some of the RYR1 variants described here have also been found in individuals with RYR1-related myopathies. Pathogenic variants in RYR1 can cause several other inherited muscle disorders, such as central core disease, multiminicore disease, congenital fiber type disproportion, centronuclear myopathy, King-Denborough syndrome, nemaline myopathy, and congenital myopathy with cores and rods. Some of the congenital myopathies are inherited in an autosomal-dominant pattern and others in an autosomal recessive pattern. These disorders typically manifest as symptomatic myopathies and should be diagnosed and managed by a neuromuscular specialist.	X	Malignant hypertherm ia susceptiblit y	RYR1 is associated with RYR1-related myopathy (definitive), malignant hyperthermia susceptibility (definitive)	Malignant hyperthermia susceptiblity (Adult and Pediatric - Strong actionability)
CACNA1S	9	20%	X	-	X	Malignant hypertherm ia susceptibili ty	CACNA1S associated with malignant hyperthermia susceptibility (moderate)	Malignant hyperthermia susceptiblity (Adult and Pediatric - Strong actionability)
CYP2C8	8	18%	-	-	-	-	-	-
SLC6A4	7	16%	-	-	-	-	SLC6A4 is associated with autism spectrum disorder (disputed)	-
BCHE	5	11%	-	-	X	-	-	-
BDNF	5	11%	-	-	-	-	-	-
CES1	5	11%	-	-	-	-	-	-

CFTR	5	11%	X	The G551D-CFTR variant is associated with CF and pancreatic insufficiency (when a nonfunctional CFTR variant is found on the other allele) (http://www.CFTR2.org). Patients should discuss their genotype results with a CF physician and/or a genetic counselor. Furthermore, genetic counseling is important for families with children diagnosed with CF and also for families of children who are carriers for CF variants, so that they can understand their risks of CF in future pregnancies.	X	-	-	-
IFNL4	5	11%	-	-	-	-	-	-

NAT2	5	11%	X	No inherited diseases or conditions have been consistently or strongly linked to germline genetic variants in NAT2 independent of xenobiotic metabolism and response. Because NAT2 is involved in the metabolism of carcinogenic compounds, individuals with reduced NAT2 metabolism may have an increased risk of certain cancers, such as bladder cancer and lung cancer, but these risks are dependent upon the extent of carcinogen exposure. The CPIC writing group does not endorse the utility of NAT2 genotyping to reliably inform cancer risk. Recent studies suggest that human NAT2 is a novel genetic factor that influences plasma lipid and cholesterol levels and alters the risk of cardiometabolic disorders. However, the ACMG does not currently recommend return of secondary findings for NAT2 from exome or genome sequencing.	X	-	-	-
UGT1A4	5	11%	-	-	-	-	-	-
CACNA1C	4	9%	-	-	-	-	CACNA1C associated with Timothy syndrome (definitive), long QT syndrome (moderate), Brugada syndrome (disputed),	-

							short QT syndrome (disputed)	
FKBP5	4	9%	-	-	-	-	-	-
HTR1A	4	9%	-	-	-	-	-	-
ATM	3	7%	-	-	X	-	ATM associated with ataxia telangiectasia (definitive) and ATM-related cancer predisposition (definitive)	Breast cancer (Adult - Moderate actionability, Pediatric- Insufficient evidence)
DBH	3	7%	-	-	-	-	-	-
GRIK1	3	7%	-	-	-	-	-	-
MC4R	3	7%	-	-	-	-	-	-
STAC3	3	7%	-	-	-	-	STAC3 is associated with Bailey-Bloch congenital myopathy (definitive)	-
ANK3	2	5%	-	-	-	-	ANK3 associated witn intellectual disability (moderate)	-
DRD3	2	5%	-	-	-	-	-	-
EPHX1	2	5%	-	-	-	-	Limited	-
ERCC1	2	5%	-	-	-	-	-	-

GGCX	2	5%	-	-	-	-	GGCX is associated with vitamin K-dependent clotting factor deficiency (definitive), GGCX is associated with pulmonary arterial hypertension (moderate)	-
GNB3	2	5%	-	-	-	-	-	-
ITGB3	2	5%	-	-	-	-	ITGB3 is associated with Glanzmann thrombastheni a (definitive) and platelet-type bleeding disorder (definitive)	Glanzmann thrombasthenia (Adult and Pediatric - Strong actionability)
KIF6	2	5%	-	-	-	-	-	-

MT-RNR1	2	5%	X	Due to the mitochondrial inheritance pattern of MT-RNR1, the identification of a clinically relevant MT-RNR1 variant in an individual will be of relevance to any of their maternal relatives (i.e. mother, siblings, mother's siblings and maternal grandmother) and to all of the children of a female identified to carry the variant. This should be communicated to the patient when a clinically relevant genotype is identified and the advice to avoid aminoglycosides should be cascaded to the relevant individuals within the family. Advice from a clinical genetics service can be sought to support the cascading of information within the family	X	-	MT-RNR1 associated with mitochondrial disease (definitive)	-
SCN1A	2	5%	-	-	-	-	SCN1A is associated with Dravet syndrome (definitive), generalized epilepsy with febrile seizures (definitive), genetic developmental and epileptic encephalopath y (definitive) and familial hemiplegic migraine (moderate)	Dravet syndrome (Adult-Insufficient evidence, Pediatric-Moderate actionability)

SLC6A2	2	5%	-	-	-	-	-	-
UGT2B7	2	5%	-	-	-	-	-	-
ABCC2	1	2%	-	-	-	-	-	-
ABCC3	1	2%	-	-	-	-	-	-
ACE	1	2%	-	-	-	-	ACE associated with renal tubular dysgenesis (definitive)	-
ACYP2	1	2%	-	-	-	-	-	-
ADHB1	1	2%	-	-	-	-	-	-
ADRB2	1	2%	-	-	-	-	-	-
AGT	1	2%	-	-	-	-	-	-
ALDH2	1	2%	-	-	-	-	-	-
ATIC	1	2%	-	-	-	-	-	-
C11orf65	1	2%	-	-	-	-	-	-
CACNG2 rs2283967	1	2%	-	-	-	-	Limited	-
CEP72	1	2%	-	-	-	-	-	-
CHRNA3	1	2%	-	-	-	-	-	-
CNR1 rs806380	1	2%	-	-	-	-	-	-
CYP1A1	1	2%	-	-	-	-	-	-
CYP2A6	1	2%	-	-	-	-	-	-
DRB1 (rs4532)	1	2%	-	-	-	-	-	-
DRD1	1	2%	-	-	-	-	-	-
EDN1	1	2%	-	-	-	-	Limited	-
F13A1	1	2%	-	-	-	-	F13A deficiency is associated with thrombosis disorder (definitive)	-
FAAH	1	2%	-	-	-	-	-	-

FCGR3A	1	2%	-	-	-	-	-	-
GABRP	1	2%	-	-	-	-	-	-
GLP1R	1	2%	-	-	-	-	-	-
GRIN2B	1	2%	-	-	-	-	GRIN2B is associated with complex neurodevelop mental disorder (definitive)	-
GRK4	1	2%	-	-	-	-	-	-
GSTP1	1	2%	-	-	-	-	-	-
*HLA-B*38:01*	1	2%	-	-	-	-	-	-
HLA-DQA1	1	2%	-	-	X	-	-	-
HLA-DQB1	1	2%	-	-	-	-	-	-
HLA-DRB1	1	2%	-	-	X	-	-	-
HTR7 rs7905446	1	2%	-	-	-	-	-	-
Inc RNA	1	2%	-	-	-	-	-	-
INSIG2 rS17047764	1	2%	-	-	-	-	-	-
ITPA	1	2%	-	-	-	-	ITPA is associated with genetic developmental and epileptic encephalopath y (definitive)	-
KCNIP1	1	2%	-	-	-	-	-	-

LDLR	1	2%	-	-	-	Familial hypercholesterolemia	LDLR is associated with familial hypercholesterolemia (definitive)	Heterozygous familial hypercholesterolemia (Adult - Definitive actionability, Pediatric - Strong actionability). Homozygous familiar hypercholesterolemia (Adult and Pediatric - Strong actionability)
NOS3	1	2%	-	-	-	-	-	-
NQO1	1	2%	-	-	-	-	-	-
NR1H3	1	2%	-	-	-	-	-	-
OPRD1 (rs2236861) (rs678849)	1	2%	-	-	-	-	-	-
OPRK1 (rs1051660) (rs6473797)	1	2%	-	-	-	-	-	-
PNPLA5	1	2%	-	-	-	-	-	-
POR rs2868177	1	2%	-	-	-	-	POR is associated with Antley-Bixler syndrome with genital anomalies and disordered steroidogenesis (definitive)	-
RARG	1	2%	-	-	-	-	-	-
SLC19A1 rs1051266	1	2%	-	-	-	-	Limited	-
SLC1A1	1	2%	-	-	-	-	Limited	-
SLC1A2	1	2%	-	-	-	-	SLC1A2 is associated with developmental	-

							and epileptic encephalopathy (definitive)	
SLC1B3	1	2%	-	-	-	-	-	-
SLC28A3	1	2%	-	-	-	-	-	-
SLC47A2	1	2%	-	-	-	-	-	-
SLC6A5	1	2%	-	-	-	-	-	-
SULT4A1	1	2%	-	-	-	-	-	-
TH (rs2070762)	1	2%	-	-	-	-	TH is associated with tyrosine hydroxylase deficiency (definitive)	-
TPH2 (rs1487278)	1	2%	-	-	-	-	-	-
TXNRD2	1	2%	-	-	-	-	-	-
UGT1A6	1	2%	-	-	-	-	-	-
XRCC1	1	2%	-	-	-	-	-	-

ACMG: American College of Medical Genetics and Genomics; ClinGen: Clinical Genome Resource; CPIC: Clinical Pharmacogenetics Implementation Consortium; DPWG: Dutch Pharmacogenetics Working Group; FDA: Food and Drug Administration, IF: Incidental Finding; SF: Secondary Finding; PGx: Pharmacogenomics
